# Comparison of CT and MRI in Diagnosis of Laryngeal Carcinoma with Anterior Vocal Commissure Involvement

**DOI:** 10.1038/srep30353

**Published:** 2016-08-02

**Authors:** Jian-hui Wu, Jing Zhao, Zeng-hong Li, Wei-qiang Yang, Qi-hong Liu, Zhi-yun Yang, Bing Liao, Xiao-ling Li, Bin Wang, Hao Qin, Jie Luo, Ke-xing Lv, Wei-ping Wen, Wen-bin Lei

**Affiliations:** 1 Otorhinolaryngology Institute at Otorhinolaryngology Hospital, The First Affiliated Hospital of Sun Yat-sen University, Guangzhou 510080, P.R. China; 2 The Otolaryngological Department, Meizhou People’s Hospital, Meizhou, Guangdong, P.R. China; 3 The Otolaryngological Department, Foshan People’s Hospital, Foshan, Guangdong, P.R. China; 4 The Otolaryngological Department, Peking University Shenzhen Hospital, Shenzhen, Guangdong, P.R. China; 5 The Otolaryngological Department, Futian People’s Hospital, Shenzhen, Guangdong, P.R. China; 6 Department of Radiology, The First Affiliated Hospital of Sun Yat-sen University, Guangzhou 510080, P.R. China; 7 Department of Pathology, The First Affiliated Hospital of Sun Yat-sen University, Guangzhou 510080, P.R. China; 8 The Fourth People’s Hospital of Shenzhen(Futian Hospital)Affiliated Futian Hospital of Guangdong Medical College, ShenZhen, Guangdong, P.R. China

## Abstract

This study aimed to compare the accuracy of CT and MRI in determining the invasion of thyroid cartilage by and the T staging of laryngeal carcinoma with anterior vocal commissure (AVC) involvement. A total of 26 cases of laryngeal carcinomas with AVC involvement from May 2012 to January 2014 underwent enhanced CT and MRI scan, out of whom 6 patients also underwent diffusion-weighted magnetic resonance imaging(DWI). T staging and thyroid cartilage involvement were evaluated. All the surgical specimens underwent serial section and were reviewed by two senior pathologists independently. When compared with pathologic staging, the accuracy was 88.46% (23/26) of MRI scan (with a 95% confidence interval 37~77%) and 57.69% (15/26) of CT scan (with a 95% confidence interval 70~98%), respectively (*P* < 0.01). We also reported three cases who were misdiagnosed on CT or MRI about either the thyroid cartilage was involved or not, and one case of preliminary study of DWI. Compared to CT, MRI exhibited a higher accuracy rate on T staging of laryngeal carcinomas with AVC involvement. Combined utility of CT and MRI could help improve the accuracy of assessment of thyroid cartilage involvement and T staging of laryngeal carcinomas with AVC involvement.

Anterior vocal commissure (AVC), considered as the transition area between supraglottic and subglottic regions, lies anteriorly between the vocal cords^1^,^2^. Approximately 20% of glottic carcinomas involve AVC, while only 1% of the tumors originate from this area^3^.Some studies reported that the malignancies could easily extend to the subglottis, thyroid cartilage and preepiglottic space through AVC^4^,^5^. However, there are some other studies illustrating that Broyles’ ligament itself can act as a barrier to protect the thyroid cartilage from tumor invasion^6^. The special anatomic characteristics of AVC have brought up challenges in staging and treating tumors involving or arising from this laryngeal subregion more difficult to stage.

Used in combination with clinical history and laryngoscopy, the accuracy rates of CT and MRI in T staging of laryngeal carcinoma could reach 80% and 87%, respectively^7^. However, in the assessment of anterior commissure lesions, MRI had a relatively high accuracy but low specificity^8^ compared with CT which either overestimates or underestimates the involvement of cartilage^9^.

Transoral carbon dioxide (CO_2_) laser surgery is a good therapeutic strategy for early staged glottic carcinomas^10^,^11^,^12^. However, when it comes to AVC involvement, transoral CO_2_ laser surgery is usually followed by a higher recurrence rate than open surgery is^13^,^14^, mainly because of difficulties in (1) preoperative assessement of thyroid cartilage involvement, (2) intraoperative exposure of AVC itself, and (3) en-bloc resection of the suspectedly involved thyroid cartilage. Thus, accurate pre-treatment assessment of AVC and thyroid cartilage involvement is the key point to avoid improper treatment planning and insufficient treatment. The objective of this study is to compare the diagnostic values of CT and MRI in assessing thyroid cartilage invasion by laryngeal carcinomas with AVC involvement, so that better treatment planning can be made.

## Materials and Methods

### Ethics Statement

The research protocols were approved by the Ethics Committee of the First Affiliated Hospital of Sun Yat-sen University, Guangdong, China ([2013]160#). The study were carried out in the accordance with the approved guidelines. Written informed consent was obtained from each patient, and all patients granted us permission to use the data obtained in subsequent studies.

### Clinical Data

Twenty-six patients who had been clinicopathologically diagnosed with laryngeal squamous cell carcinoma (SCC) with AVC involvement were treated in the Department of otolaryngology, the first affiliated hospital of Sun Yat-sen university between May 2012 and January 2014 were enrolled in our study. None of these patients had had any previous surgery, radiotherapy or chemotherapy before being hospitalized in our department. All the surgical samples were re-confirmed by postoperative pathology.

### Imaging Examination and Analysis

Each included patient accepted both plain and enhanced CT and MRI scans. CT scans were obtained with a 64-slice spiral CT scanner (Toshiba, Japan). Iopromide was used as the contrast medium. Axial (0.5–1 mm), sagittal (3–5 mm) and coronal (3–5 mm) images were reconstructed with both soft tissue and bone algorithms. MRI scans were obtained with a 3.0 Tesla(T) MRI scanner (Trio, Siemens, Germany). The field of view (FOV) was 230 × 230 × 137 mm, with acquisition matrix 512 × 512. The scans were performed before and after the intravenous administration of magnetic meglumine, following the fast spin-echo sequence (SE) in T1WI (TR/TE400–600/10–15 ms), and in T2WI (TR/TE 3500–6000/100–150 ms). The scan slices were 3–4 mm in thickness without interuptions. Six paitents also underwent diffusion-weighted magnetic resonance imaging (DWI) before enhanced MRI scanning, calculating apparent diffusion coefficient (ADC) values with b-values of 0 and 800 s/mm^2^.

The CT and MRI images were analyzed independently by two senior radiologists. The two film readers were blinded to the pathological results. The site and extension of lesion, signs of impaired vocal cord mobility and deep laryngeal invasion (paraglottic or preepiglottic spaces and thyroid cartilage) were assessed. On CT images, the invasion of thyroid cartilage was defined based on the disappearance of fat space between the tumor and the cartilage, or destruction of cartilage or bone cortex. On MRI images, cartilage invasion by the tumor was considered when the cartilage had similar signal intensity to that of the adjacent tumor mass on T2WI or similar enhancement was seen in the cartilage to that of the adjacent tumor. The final results were obtained after the two radiologists got a consensus opinion through a final discussion, according to which radiographic T stages (UICC-2002 TNM) were classified (cT: radiographic stage by CT; mT: radiographic stage by MRI).

### Surgery and Postoperative Pathology

All the patients underwent transcervical open surgeries, including 8 total laryngectomies, 8 frontal partial laryngectomies and 10 supracricoid partial laryngectomies. The sugical specimens were resected integrally and then fixed with 10% formalin. After that, the specimens were cut into 3–6 segments parallel to the plane of vocal cords and then paraffin-embedded. Each embedded segments were further cut into axial serial section slices (5 μm) parallel to the plane of vocal cords at 0.4 mm intervals. Hematoxylin-eosin staining was performed on each slice.

All the slices were reviewed independently by two senior pathologists. The final results were obtained after the two pathologists reached a consensus through a final discussion, according to which pathological T staging (pT) was classified. Sections were scanned and saved by a digital slice scanner (nanozoomer 2.0HT, Japan).

### Statistical Analysis

Statistical analysis was performed with SPSS V13.0. The difference of accuracy was calculated using *Χ*^2^ test, with *p* value < 0.05 considered statistically significant.

## Results

### Patient characteristics

All the 26 patients were male, with a median age of 61.5 years (46–81 years). Smokers represented 89.40% of the patients, whereas 78.39% were alcohol consumers, and association between tobacco and alcohol was present in 74.48% of cases.

The pathological T stages included 10 (38.46%) pT2, 10 (38.46%) pT3 and 6 (15.79%) pT4. Involvement of thyroid cartilage was found in 11 of the 26 cases (42.31%).Seven out of the 10 pT2 patients had limited vocal cord mobility.

### Accuracy of Imaging

According to pT staging, the T staging accuracies of CT and MRI were 57.69% (15/26) (95% CI 37~77%, Table 1) and 88.46% (23/26) (95% CI 70~89%, Table 2), respectively, where a significant difference was revealed by McNemar’s test (*P* < 0.01).

### Case Reports

CT and MRI exhibited their differences in determining local infiltration and tumor extension. Four cases were selected to be elucidated as follows.

### Case 1 Involvement of Thyroid Cartilage without Invading into the Paraglottic Space

**Figure 1:**
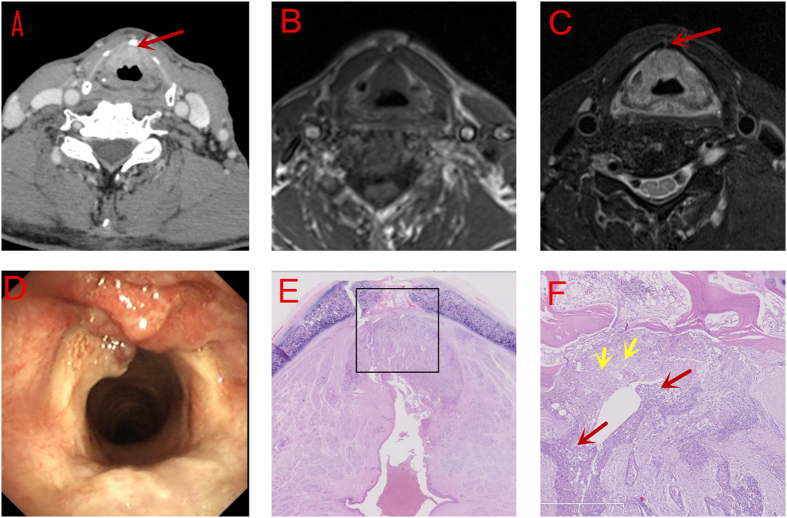
Involvement of Thyroid Cartilage without Invading into the Paraglottic Space. (**A**) CT scan: without involvement of thyroid cartilage (red arrow) (**B**) (T1WI)and (**C**) (T2WI, fat-suppression) MRI scan: Involvement of thyroid cartilage (red arrow) (**D**). Electronic laryngoscopic examination. (**E**,**F**) (amplified image within the rectangle in (**E**), 200×) Serial section: mild invasion of the thyroid cartilage (Red arrow: tumor cells; Yellow arrow: the impaired thyroid cartilage).

MRI revealed that the tumor was located at the anterior part of both vocal cords and the AVC, with an extension to the supraglottis including the lower epiglottis. Axial MRI images revealed that the tumor invaded the thyroid cartilage without paraglottic space (PGS) involvement (Fig. 1B,C). The judement of tumor location and extension on CT was similar to that on MRI, except for that the definite destruction of cartilage was not observed on CT (Fig. 1A). Pathological examination proved that the tumor had invaded the thyroid cartilage Fig. 1E,F).

### Case 2 PGS Invasion without Thyroid Cartilage Involvement

**Figure 2:**
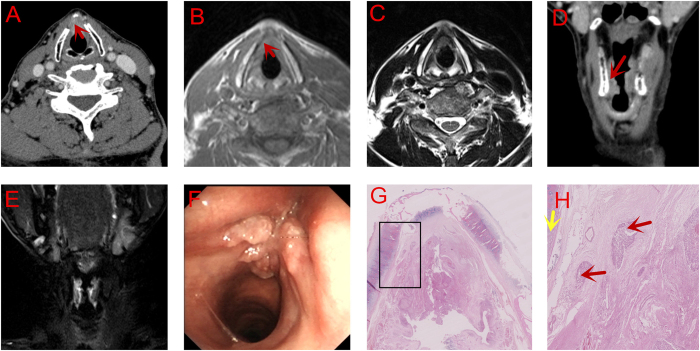
PGS Invasion without Thyroid Cartilage Involvement. (**A**) (Axial) and (**D**). (Coronal) CT scan: Involvement of thyroid cartilage (red arrow). (**B**) (Enhanced T1WI, Axial), (**C**). (T2WI, Axial) and E. (T2WI, Coronal) MRI scan: Without involvement of thyroid cartilage. (**F**) Electronic laryngoscopic examination. (**G**,H) (amplified image within the rectangle in G., 40 ×) Serial section: Without involvement of thyroid cartilage (Red arrow: tumor cells; Yellow arrow: thyroid cartilage).

CT revealed that the tumor involved both the vocal cords, extending superiorly to the ventricular cords and inferiorly to the subglottis. Additional to an unevenly enhanced density of the lesion on enhanced CT, an unclear margin was also seen between the tumor and thyroid cartilage at the left glottic region, demonstrating that the tumor had involved the cartilage (Fig. 2A,D). The similar judgement of tumor extension was obtained on MRI. However, the hypointensity signal of PGS on T1WI at the level of vocal cord implied that the left PGS had been invaded by the tumor. The extension of tumor, involvement of the thyroid cartilage and left PGS were all confirmed by pathology. On the other hand, the thyroid cartilage was not found to be involved either on MRI (Fig. 2B,C,E) or by postoperative pathological examination (Fig. 2G,H).

### Case 3 Thyroid Cartilage Uninvaded

**Figure 3:**
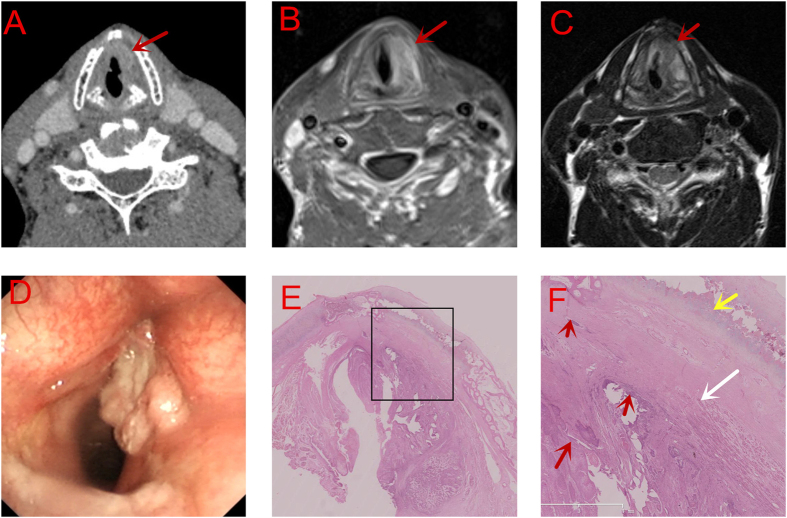
Thyroid Cartilage Uninvaded. (**A**) CT scan: Without involvement of thyroid cartilage (red arrow) (**B**).(T1WI)and (**C**).(T2WI) MRI scan: Involvement of thyroid cartilage (red arrow) (**D**). Electronic laryngoscopic examination. (**E**,**F**) (amplified image within the rectangle in E., 40×) Serial section: Without involvement of thyroid cartilage (Red arrow: tumor cells; Yellow arrow: the thyroid cartilage ;White arrow: the vocal muscle).

CT revealed that the tumor involved both the vocal cords and extended superiorly to the ventricular cords and inferiorly to the subglottis. The lesion exhibited an enhanced density and a clear margin from the ossification of thyroid cartilage (Fig. 3A). MRI confirmed the tumor extension seen on CT. However, hypointensity on T1WI and uneven intensity on T2WI of the right thyroid cartilage depicted that it had been invaded by the tumor (Fig. 3B,C). However, it was confirmed by postoperative pathological examination that the thyroid cartilage was still intact (Fig. 3E,F).

### Case 4 The Preliminary Study of DWI

**Figure 4:**
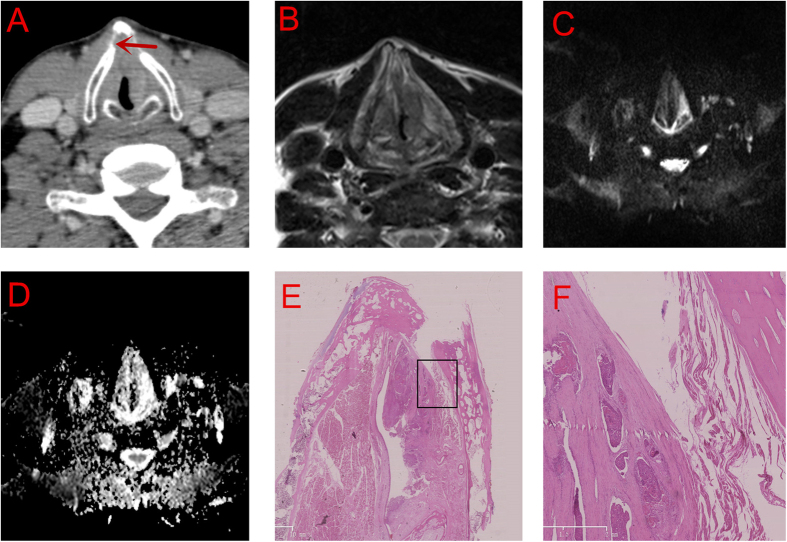
The preliminary study of DWI. (**A**) CT scan: suspected perichondrium invasion. (**B**) (T2WI) (**C**) (DWI) and (**D**) (ADC) MRI scan: Without involvement of perichondrium. (**E**,**F**) (amplified image within the rectangle in E., 200×) Serial section: Without involvement of perichondrium.

CT revealed that the tumor was adjacent to the thyroid cartilage, suggesting suspected invasion of the perichondrium (Fig. 4A), which was, however, demonstrated clearly intact on MRI (Fig. 4B). The DWI signal (Fig. 4C) and ADC (Fig. 4D) of the tumor were slightly higher than and equivalent to, respectively, those of the muscles.

## Discussion

Therapeutic strategies of laryngeal carcinomas with AVC involvement are still controversial. Radiotherapy is less effective in treating this area^15^. Surgery by transcervical approaches with resection of suspectedly invaded cartilage is recommended to avoid relapse^14^. Despite the guarantee of low recurrence rate, the conservative surgical strategy brings many sequelae and side effects^16^. Transoral CO_2_ laser surgery has many advantages in treating early-staged cases, including avoidance of tracheotomy, shorter hospital stay, less cost, better voice quality and fewer postoperative complications compared with organ preservation surgery^4^,^10^. But accurate assessment of tumor invasion for optimized treatment planning is still fraught with challenges.

The advantages and disadvantages of CT and MRI in the pre-operative assessment of laryngeal carcinoma still need to be further studied. On one hand, the involvement of cartilage may be overestimated in MRI leading to overtreatment, or underestimated in CT leading to inadequate treatment^8^,^9^. Therefore, the option of CT or MRI in practice is more dependent on the performance of the machines and the doctors’ preferences^17^. On the other hand, there are very few reports on the “features” of tumor invasion to the anterior commissure and thyroid cartilage in CT or MRI; and the value of DWI used in the assessment of laryngeal carcinoma also needs further exploration^18^.

CT scan has been used for preoperative evaluation on AVC tumors. Barbosa *et al*.^19^ reported an accuracy of 75% of spiral CT scan for AVC tumors. suggesting an important role CT played in T staging. However, Hart *et al*.^20^ reported that spiral CT scan could not precisely reveal the invasion of thyroid cartilage, which might have been due to the impaired mobility of vocal cords caused by AVC involvement. In the first case of our study, the tiny glottic tumor invading the thyroid cartilage was identified on MRI but NOT on CT scan, presumably due to the limitation of its resolution, leading to a misdiagnosis (Fig. 1). The involvement of cartilage can be classified by CT as follows: sclerosis, erosion, lysis and extralaryngeal extension^21^. However, it may be difficult to distinguish these signs in case of mild infiltration of the inner cortex of the cartilage^22^,^23^. Moreover, the low resolusion of CT on distinguishing soft tissue also limited its accuracy in the evaluation of the paraglottic space. In our Case 2, CT detected the invasion of thyroid cartilage but missed the invasion of PGS which was later evidenced on MRI scanning. This could be explained by the poor resolution of CT on distiguishing soft tissues (Fig. 2).

Our study revealed that MRI was more accurate than CT scan in T staging (*P* < 0.01) (Table 3). Compared with CT, MRI showed a higher sensitivity (93~96%) and a higher accuracy (84~76%) in detecting AVC and thyroid cartilage involvement, echoing with some other studies^24^,^25^,^26^. However, the specificity of MRI was low^27^, which was considered to be correlated with local inflammation caused by tumors. Besides, the results of MRI, especially DWI scan(Fig. 4C,D), is more likely to be influenced by examination time and movements such as breathing and swallowing. For patients with obstructive dyspnea or low compliance, large artifacts could be seen on MRI, causing incorrect judgement of tumor extension. as was seen in our third case (Fig. 3). Additionally, the slice thickness of MRI is another disadvantage of it.

Some previous studies have proposed a series of signs or features on CT or MRI to diagnose AVC tumors or AVC involvement by tumors. Barbosa *et al*.^19^ summarized a sign of gross radiologic anterior commissure involvement (GRACI) to determine T stage of AVC tumors, with an accuracy from 75% to over 96% on CT. Moreover, the thickness and demension of reconstructed slices must also be emphasized on CT. The routine thickness is 1–2 mm, which often cause “skip” of valuable signs with the laryngx. Hence, a thickness of 0.5 mm is recommended for laryngeal tumors. In addition to axial and coronal reconstruction of slices, saggital slices are also recommanded in the assessment of AVC. For MRI, the intensity features of a tumor and inflammation should be distingushed with caution. According to Becker *et al*.^28^, inflammation lesions often have higher signals than tumors on both T2WI and enhanced T1WI. In our study, in order to distinguish tumors from inflammation and to stage the primary tumor, we used DWI scan on six cases, which is believed to able to reveal the microcirculation and water molecule diffusion patterns within the tumor^29^. Mohamed S Taha *et al*.^30^ reported that DWI showed high validity and precision in detecting inner and outer thyroid lamina invasion, which helped in the decision of management of laryngeal carcinoma. DWI may also detect changes in tumor size and shape before they are visible by laryngostroboscopy^31^, facilitating early diagnosis. Besides, a series of techniques such as signal to noise ratio (SNR) and contrast to noise ratio (CNR) have been developed and used in 3.0T MRI scanning, promising a higher sensitivity on lesion detection. However, this higher sensitivity may also cause difficulties in the distinguising tumor and inflammation. Also, even a slight movement during the scanning can generate great motion artifacts^32^,^33^,^34^.

Besides density on CT and signal intensity on MRI, the contour of lesions should also be carefully judged. For example, a small convex protruding into the cartilage, as is seen in Fig. 1C, should suggest cartilage invasion by the tumor. While a mass lesion compressing the adjacent cartilage, as is seen in Fig. 2A, with good continuity of the chondrial cortex, may exclude cartilage involvement by the tumor. These features are useful in the determination of cartilage involvement and thus treatment planning, particularly for T3 lesions. Besides, clinicians should be aware of the importance of serial imaging reading on the high definition display. Thicker reconstruction slices on MRI than on CT is more likely to skip some tiny but valuable signs. The best b-value also needs to be further studied on DWI scan. However, as the technology develops, it is hopefully that MRI will provide more precise assessment of tumor extension^27^. Besides, we advise that more features should be explored both on CT and MRI.

## Conclusion

In conclusion, MRI exhibited a higher accuracy than CT on T staging of laryngeal carcinomas with AVC involvement. However, each of the two imaging techniques has their own advantages and disadvantages on determining the local infiltration and extension of tumors. Combined utility of CT and MRI could help overcome the disadvantages and improve the accuracy of thyroid cartilage assessment and T staging of laryngeal carcinomas with AVC involvement.

## Additional Information

**How to cite this article**: Wu, J.-H. *et al*. Comparison of CT and MRI in Diagnosis of Laryngeal Carcinoma with Anterior Vocal Commissure Involvement. *Sci. Rep.* **6**, 30353; doi: 10.1038/srep30353 (2016).

## Figures and Tables

**Table 1:** T stage comparison between CT and pathologic stage.

cT	pT				
	T1	T2	T3	T4	Total
T1	0	3	0	0	3
T2	0	5	5	1	11
T3	0	2	5	0	7
T4	0	0	0	5	5
Total	0	10	10	6	26

cT: radiographic stage by CT. pT: pathological T staging.

**Table 2:** T stage comparison between MRI and pathologic stage.

mT	pT				
	T1	T2	T3	T4	Total
T1	0	1	0	0	1
T2	0	9	2	0	11
T3	0	0	8	0	8
T4	0	0	0	6	6
Total	0	10	10	6	26

mT: radiographic stage by MRI. pT: pathological T staging.

**Table 3:** Comparison of the accuracy of CT and MRI on T staging based on pathological findings.

CT	MRI		*P*
	+	−	
+	15	0	
−	8	3	<0.01
Total	23	3	

Statistically significant with *P* < 0.05, McNemar’s test. +accurate, −misdiagnose.
